# Efficacy and safety of leuprorelin (Boennuokang^®^) plus endocrine therapy in premenopausal women with HR^+^/HER2^−^ breast cancer

**DOI:** 10.3389/fphar.2025.1594799

**Published:** 2025-07-03

**Authors:** Xiuping Wu, Yinfeng Lin, Wangmei Xie, Yan Jiang, Zhenchao Xu, Kai He, Shuyu Li

**Affiliations:** Department of Breast Surgery, Zhangzhou Zhengxing Hospital, Zhangzhou, China

**Keywords:** hormone receptor-positive/human epidermal growth factor receptor 2-negative breast cancer, premenopausal women, leuprorelin (Boennuokang ^®^), efficacy, safety

## Abstract

**Background:**

Leuprorelin shows good efficacy in premenopausal women with hormone receptor-positive (HR^+^)/human epidermal growth factor receptor 2-negative (HER2^−^) breast cancer. However, more real-world evidence is required. This real-world study aimed to explore the efficacy and safety of leuprorelin (Boennuokang^®^) plus endocrine therapy in premenopausal women with HR^+^/HER2^−^ breast cancer.

**Methods:**

A total of 229 premenopausal women with HR^+^/HER2^−^ breast cancer receiving adjuvant leuprorelin plus endocrine therapy were included in this retrospective study. Leuprorelin (Boennuokang^®^) was administered 3.75 mg subcutaneously every 28 days following surgery. Endocrine therapy contained aromatase inhibitors, selective estrogen receptor modulators, and selective estrogen receptor degraders. The median follow-up duration of this study was 38.1 months.

**Results:**

The estradiol (E2) level was declined from 46.0 to 19.0 pg/mL over 24 months (*P* < 0.001). E2 from month 3 to month 24 was maintained below 30 pg/mL (menopausal level). During 24 months, the follicle-stimulating hormone level was decreased from 7.7 to 4.8 mIU/mL, and the luteinizing hormone level was decreased from 7.9 to 0.2 mIU/mL (both *P* < 0.001). During the follow-up period, 9 patients experienced disease recurrence. The 10-year accumulating progression-free survival (PFS) rate was 91.7%. Comorbidity (yes vs no) was independently related to shorter progression-free survival (hazard ratio: 10.957, *P* = 0.003). Bone soreness (6.1%) was the most common adverse event, followed by hot flushes (3.5%), morning stiffness (1.3%), and muscle soreness (1.3%).

**Conclusion:**

Leuprorelin (Boennuokang^®^) plus endocrine therapy reduces gonadotropins and sex hormones and results in satisfactory survival rates with good safety profiles in premenopausal women with HR^+^/HER2^−^ breast cancer.

## 1 Introduction

Breast cancer is the most common cancer in females, accounting for approximately 2.3 million new cases in 2022 (Bray et al., 2024). Hormone receptor-positive (HR^+^)/human epidermal growth factor receptor 2-negative (HER2^−^) represents the most common molecular subtype of breast cancer (Giaquinto et al., 2022; Jin et al., 2023). In premenopausal women with HR^+^/HER2^−^ breast cancer, ovarian function plays a critical role in driving tumor progression through the production of female hormones (Cao et al., 2024; Corti et al., 2023; Francis, 2023). To reduce the risk of postoperative tumor recurrence and improve survival, adjuvant endocrine therapy is the recommended treatment to suppress estrogen production in premenopausal women with HR^+^/HER2^−^ breast cancer (Burstein et al., 2019; Corti et al., 2023; Cucciniello et al., 2023; Lopez-Tarruella et al., 2022).

Ovarian function suppression (OFS) is a key strategy in endocrine therapy (Arboleda et al., 2022; Brett and Mayer, 2023; Glassman et al., 2017; Scharl and Salterberg, 2016). OFS inhibits estrogen production to mitigate the facilitating effects of estrogen on tumor cells, which can be achieved through several surgical and medical methods (Lu et al., 2021). The guidelines suggest that OFS should be considered in high-risk premenopausal women with HR^+^/HER2^−^ breast cancer (The Society of Breast Cancer China Anti-Cancer Association Burstein et al., 2019; Gradishar et al., 2024). Leuprorelin, a gonadotropin-releasing hormone (GnRH) agonist, is one of the most commonly used agents for OFS (Kelly et al., 2024; Lu et al., 2021). It acts by continuously stimulating the pituitary gland, leading to downregulation of gonadotropin secretion and subsequent suppression of ovarian estrogen production (Lu et al., 2021). Previous studies have reported that leuprorelin shows good efficacy in premenopausal women with HR^+^ breast cancer (Kurebayashi et al., 2017; Lee et al., 2020; Wu et al., 2021).

Leuprorelin (Boennuokang^®^), a generic drug produced by Beijing Biote Pharmaceutical Co., Ltd., shows satisfactory efficacy in the treatment of prostate cancer (Zhou et al., 2024). Preclinical toxicology studies showed that the LD_50_ of leuprorelin (Boennuokang^®^) was >3,000 mg/kg for subcutaneous injection in mice and >100 mg/kg for intravenous injection in dogs. However, its efficacy and safety in premenopausal women with HR^+^/HER2^−^ breast cancer under real-world conditions require further exploration.

Accordingly, the current real-world study aimed to explore the efficacy and safety of leuprorelin (Boennuokang^®^) plus endocrine therapy in premenopausal women with HR^+^/HER2^−^ breast cancer.

## 2 Methods

### 2.1 Patients

This was a retrospective, single-center, single-arm study to assess the effects of adjuvant leuprorelin in premenopausal women with HR^+^/HER2^−^ breast cancer. This study began in January 2015 and ended in December 2023. The inclusion criteria for the study were as follows: 1) confirmed diagnosis of breast cancer per pathological method; 2) ≥18 years of age at breast cancer diagnosis; 3) premenopausal status; 4) received adjuvant OFS plus endocrine therapy; and 5) HR^+^ status confirmed by immunohistochemistry (IHC) of the surgical specimen. The exclusion criteria were as follows: 1) HER2-positive (HER2^+^) status confirmed by IHC; 2) history of bilateral oophorectomy, ovarian radiation, pituitary surgery, adrenalectomy, or pituitary abnormalities; 3) other primary malignancies; and 4) prior or ongoing use of long-term hormonal therapy, like oestrogen and progestogen therapy, that could interfere with ovarian function or estrogen levels. The typical IHC images for HR^+^/HER2^−^ and HER2^+^ were shown in Supplementary Figures S1 and S2, respectively. The patients were consecutively enrolled in this study. The Ethics Committee approved this study.

### 2.2 Data collection

Data were retrospectively collected from the medical records of the eligible patients. The following demographic and clinical data were extracted: age, body mass index (BMI), comorbidities, history of radiotherapy, pathological type, lesion side, pathological T-N-M stages, number of pregnancies, number of deliveries, risk stratification, and endocrine concomitant medications. Additionally, laboratory data, including E2, follicle-stimulating hormone (FSH), and luteinizing hormone (LH) levels at baseline and 3, 6, 12, 18, and 24 months after baseline (M3, M6, M12, M18, and M24), were retrospectively screened. In addition, the disease progression state with the corresponding time was collected to determine the accumulating progression-free survival (PFS) rate. Adverse events were assessed according to the National Cancer Institute Common Terminology Criteria for Adverse Events (version 5.0).

### 2.3 Treatment information

All included patients received adjuvant OFS plus endocrine therapy. Leuprorelin (Boennuokang^®^) was used for OFS, whose conventional regimen was 3.75 mg subcutaneously every 28 days following breast cancer surgery. Endocrine concomitant medications containing aromatase inhibitors (AIs), selective estrogen receptor modulators (SERMs), and selective estrogen receptor degraders (SERDs) were used for adjuvant endocrine therapy. In addition, patients may have received chemotherapy or radiotherapy, as determined by their willingness, disease status, and clinical oncologist.

### 2.4 Statistics

SPSS software (ver. 29.0, IBM, United States) was used for the data analyses. Descriptive statistics were performed, in which the median with interquartile range (IQR) was used for continuous variables, and numbers with percentages were used for classified variables. The Friedman test was used to compare E2, FSH, and LH levels over time. Additionally, subgroup analyses were performed to evaluate the changes in E2, FSH, and LH levels over time separately for different BMI categories and for patients with or without a history of radiotherapy. A Kaplan‒Meier (K-M) curve was generated to determine the accumulating PFS rate. Univariable and multivariable Cox regression analyses were conducted on PFS. The variables involved in the univariable analysis include age, BMI, comorbidity, history of radiotherapy, pathological type, lesion side, pathological T stage, pathological N stage, pathological M stage, number of pregnancies, number of deliveries, risk stratification, endocrine concomitant medications, E2, FSH, and LH. All variables involved in the univariable analysis were included in the multivariable analysis *via* a forward-stepwise method. Missing data were addressed by listwise deletion. *P* < 0.05 was considered significant.

## 3 Results

### 3.1 Clinical characteristics

A total of 33.6% of patients were aged <45 years, and the other 66.4% of patients were aged ≥45 years. Seventeen (7.4%), 145 (64.4%), and 67 (29.3%) patients carried low-, intermediate-, and high-risk, respectively. In terms of endocrine concomitant medications, 58.1%, 40.6%, and 1.3% of patients received exemestane/anastrozole/letrozole, tamoxifen/toremifene, and fulvestrant, respectively. The median (IQR) values of baseline E2, FSH, and LH were 46.0 (13.0–111.0) pg/mL, 7.7 (4.2–47.2) mIU/mL, and 7.9 (3.7–27.8) mIU/mL, respectively. The detailed clinical information is shown in Table 1.

**TABLE 1:** Clinical characteristics of premenopausal women with HR^+^/HER2^−^ breast cancer.

Characteristics	Patients (N = 229)
Age, n (%)	
<45 years	77 (33.6)
≥45 years	152 (66.4)
BMI, n (%)	
<24 kg/m^2^	128 (55.9)
≥24 kg/m^2^	101 (44.1)
Comorbidity, n (%)	
No	218 (95.2)
Yes	11 (4.8)
History of radiotherapy, n (%)	
No	61 (26.6)
Yes	168 (73.4)
Pathological type, n (%)	
Ductal	182 (79.5)
Lobular	5 (2.2)
Others	42 (18.3)
Lesion side, n (%)	
Left	111 (48.5)
Right	110 (48.0)
Both	8 (3.5)
Pathological T stage, n (%)	
T0	2 (0.9)
Tis	11 (4.8)
T1	119 (52.0)
T2	70 (30.6)
T3	6 (2.6)
T4	8 (3.5)
Unknown	13 (5.7)
Pathological N stage, n (%)	
N0	109 (47.6)
N1	77 (33.6)
N2	21 (9.2)
N3	5 (2.2)
Unknown	17 (7.4)
Pathological M stage, n (%)	
M0	211 (92.1)
M1	2 (0.9)
Mx	3 (1.3)
Unknown	13 (5.7)
Number of pregnancies, n (%)	
0	7 (3.1)
1	77 (33.6)
2	95 (41.5)
3	27 (11.8)
4 or above	23 (10.0)
Number of deliveries, n (%)	
0	8 (3.5)
1	89 (38.9)
2	115 (50.2)
3	12 (5.2)
4	3 (1.3)
Unknown	2 (0.9)
Risk stratification, n (%)	
Low risk	17 (7.4)
Intermediate risk	145 (64.4)
High risk	67 (29.3)
Endocrine concomitant medications, n (%)	
Exemestane/anastrozole/letrozole	133 (58.1)
Tamoxifen/toremifene	93 (40.6)
Fulvestrant	3 (1.3)
E2 (pg/mL), median (IQR)	46.0 (13.0–111.0)
FSH (mIU/mL), median (IQR)	7.7 (4.2–47.2)
LH (mIU/mL), median (IQR)	7.9 (3.7–27.8)
Menopausal status at follow-up, n (%)	229 (100.0)

Abbreviations: HR^+^, hormone receptor-positive; HER2^−^, human epidermal growth factor receptor 2-negative; BMI, body mass index; T, tumor; N, node; M, metastasis; E2, estradiol; IQR, interquartile range; FSH, follicle-stimulating hormone; LH, luteinizing hormone.

### 3.2 Changes in E2, FSH, and LH after leuprorelin (Boennuokang^®^) plus endocrine therapy

**FIGURE 1:**
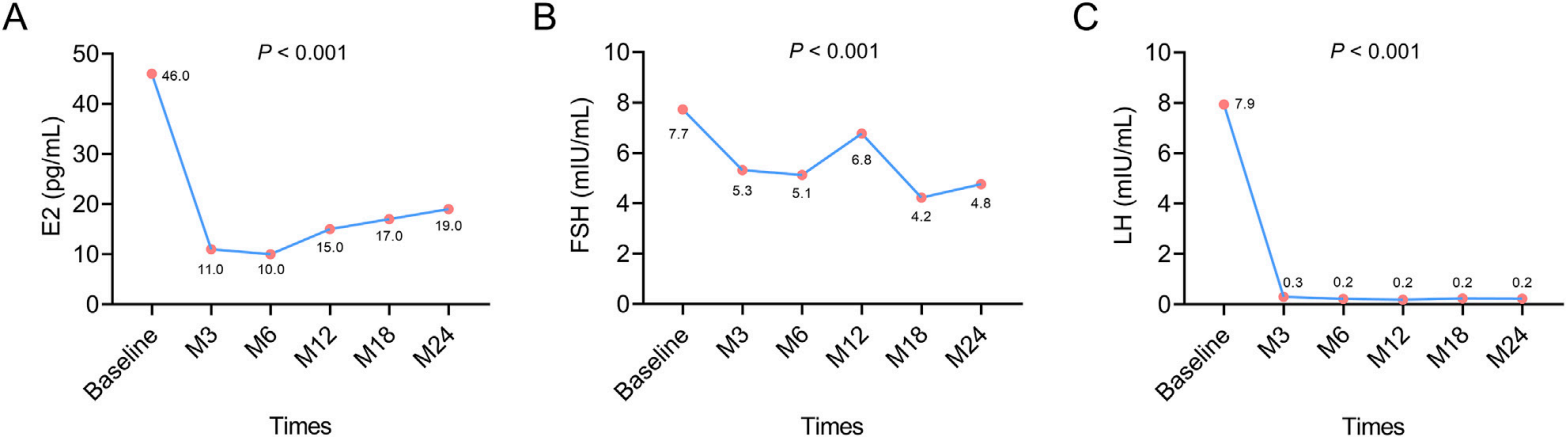
Changes in E2, FSH, and LH after leuprorelin (Boennuokang^®^) plus endocrine therapy in premenopausal women with HR^+^/HER2^−^ breast cancer. E2 **(A)**, FSH **(B)**, and LH **(C)** all exhibited a decreasing trend following leuprorelin (Boennuokang^®^) plus endocrine therapy in premenopausal women with HR^+^/HER2^−^ breast cancer.

The median E2 level was decreased from baseline (46.0 pg/mL) to M6 (10.0 pg/mL), after which its value was slightly increased from M6 to M24 (19.0 pg/mL) (*P* < 0.001). However, the rise in E2 values from M6 to M24 was not biologically meaningful because its values were maintained below 30 pg/mL, which was the menopausal level (Figure 1A). The median FSH level showed an overall decreasing trend from baseline (7.7 mIU/mL) to M24 (4.8 mIU/mL) (*P* < 0.001) (Figure 1B). The median LH level was reduced from baseline (7.9 mIU/mL) to M6 (0.2 mIU/mL), after which it remained at 0.2 mIU/mL until M24 (*P* < 0.001) (Figure 1C). The changes in E2, FSH, and LH levels from baseline to M24 suggested that leuprorelin (Boennuokang^®^) plus endocrine therapy successfully maintained the levels of E2 below the menopausal level and could suppress the production of FSH and LH.

Subgroup analyses showed that E2 and FSH exhibited overall decreasing trends during 24 months in patients with a BMI <24 kg/m^2^ (both *P* < 0.01), but these trends were not obvious in patients with a BMI ≥24 kg/m^2^ (both *P* > 0.05). LH was decreased over 24 months in both types of patients with BMI < or ≥24 kg/m^2^ (both *P* < 0.05) (Supplementary Figures S3A–C). The above findings indicated that the effect of leuprorelin (Boennuokang^®^) plus endocrine therapy on suppressing the production of sex hormones and gonadotropins might be profound in patients with a BMI <24 kg/m^2^. Regarding the history of radiotherapy, E2, FSH, and LH all exhibited decreasing trends from baseline to M24, regardless of the history of radiotherapy (all *P* < 0.05) (Supplementary Figures S3D–F). These findings disclosed that the history of radiotherapy could not affect the effect of leuprorelin (Boennuokang^®^) plus endocrine therapy on the production of sex hormones and gonadotropins.

### 3.3 PFS information

**FIGURE 2:**
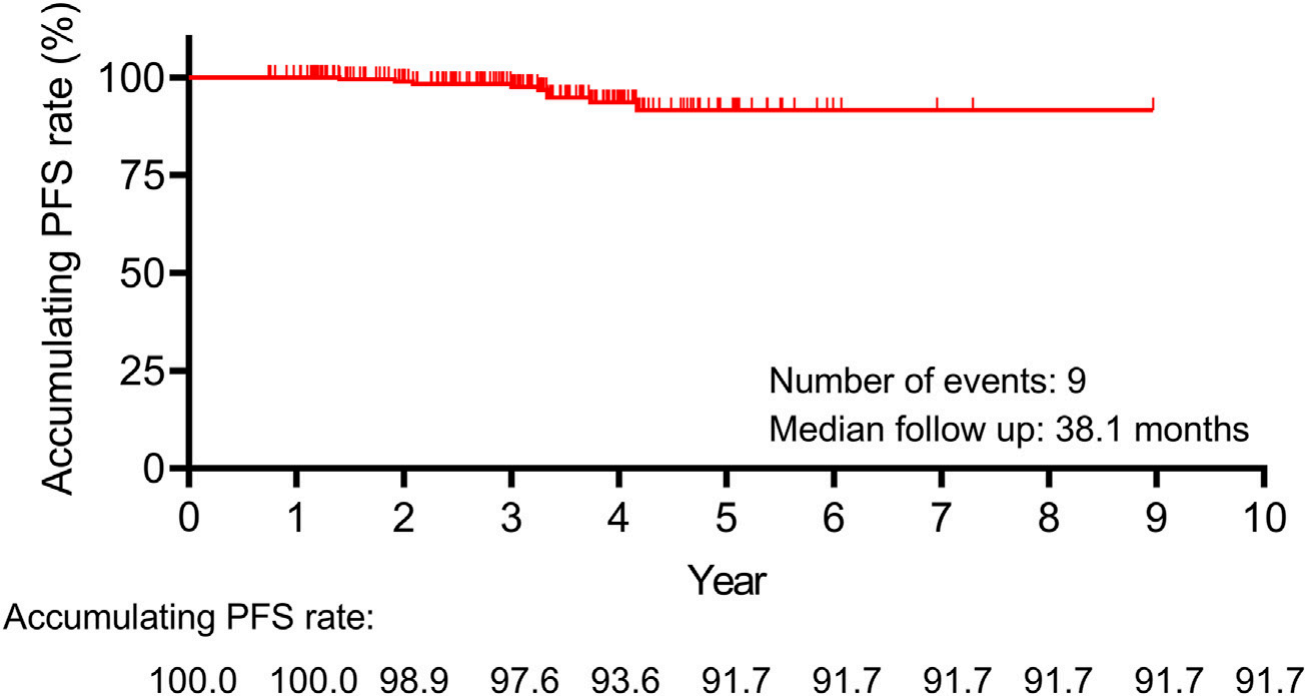
The 10-year accumulating PFS rate was 91.7% in premenopausal women with HR^+^/HER2^−^ breast cancer receiving leuprorelin (Boennuokang^®^) plus endocrine therapy.

The median follow-up duration was 38.1 months. During the follow-up period, a total of 9 patients experienced disease recurrence. The 10-year accumulating PFS rate was 91.7% (Figure 2). The above data suggested that leuprorelin (Boennuokang^®^) plus endocrine therapy could achieve a favorable prognosis in premenopausal women with HR^+^/HER2^−^ breast cancer. However, only 9 disease progression or death events occurred, which might limit the statistical power.

### 3.4 Factors related to PFS

According to the univariate Cox regression analyses, comorbidity (yes vs no) was associated with shorter PFS (hazard ratio: 13.964, *P* < 0.001). Of note, the history of radiotherapy was not related to PFS (*P* > 0.05). Additionally, PFS was not influenced by SERMs, SERDs, or AIs (*P* > 0.05). Apart from them, other factors were also not related to PFS, including age, BMI, pathological type, lesion side, pathological T stage, pathological N stage, pathological M stage, number of pregnancies, number of deliveries, risk stratification, and baseline E2, FSH, and LH. According to the multivariate Cox regression analyses, only comorbidity (yes vs no) was independently related to shorter PFS (hazard ratio: 10.957, *P* = 0.003) (Table 2). Findings from multivariate Cox regression analyses suggested that the presence of comorbidity contributed to a poor PFS in premenopausal women with HR^+^/HER2^−^ breast cancer receiving leuprorelin (Boennuokang^®^) plus endocrine therapy.

**TABLE 2:** Univariable and multivariable Cox regression analyses on PFS.

Characteristics	*P* Value	HR	95% CI	
			Lower	Upper
Univariate analyses				
Age (≥45 years vs <45 years)	0.619	1.490	0.309	7.182
BMI (≥24 kg/m^2^ vs <24 kg/m^2^)	0.140	2.840	0.709	11.378
Comorbidity (yes vs no)	<0.001	13.964	3.459	56.373
History of radiotherapy (yes vs no)	0.664	1.417	0.294	6.826
Pathological type				
Ductal vs reference	(−)	1.000	(−)	(−)
Lobular vs reference	0.825	0.837	0.173	4.042
Others (reference)	0.988	0.000	0.000	NR
Lesion side				
Left vs reference	(−)	1.000	(−)	(−)
Right vs reference	0.952	18,669.635	0.000	7.142E+143
Both (reference)	0.962	2,670.107	0.000	1.027E+143
Pathological T stage (per stage)	0.427	1.365	0.633	2.945
Pathological N stage (per stage)	0.098	1.775	0.899	3.504
Pathological M stage (per stage)	0.077	2.620	0.900	7.633
Number of pregnancies (per time)	0.279	0.631	0.274	1.453
Number of deliveries (per time)	0.611	0.774	0.288	2.077
Risk stratification (per degree)	0.962	1.028	0.323	3.270
Endocrine concomitant medications				
AIs (reference)	(−)	1.000	(−)	(−)
SERMs vs AIs	0.786	0.825	0.206	3.306
SERDs vs AIs	0.989	0.000	0.000	NR
E2 (per pg/mL)	0.959	1.000	0.995	1.005
FSH (per mIU/mL)	0.125	0.944	0.876	1.016
LH (per mIU/mL)	0.089	0.918	0.833	1.013
Multivariate analyses (forward step-wise method)				
Comorbidity (yes vs no)	0.003	10.957	2.202	54.521

Abbreviations: PFS, progression-free survival; HR, hazard ratio; CI, confidence interval; BMI, body mass index; NR, not reached; T, tumor; N, node; M, metastasis; E2, estradiol; FSH, follicle-stimulating hormone; LH, luteinizing hormone; AIs, aromatase inhibitors; SERMs, selective estrogen receptor modulators; SERDs, selective estrogen receptor degraders; AIs, included exemestane, anastrozole, and letrozole; SERMs, included tamoxifen and toremifene; SERDs, included fulvestrant.

### 3.5 Safety

Bone soreness (6.1%) was the most common adverse event, followed by hot flushes (3.5%), morning stiffness (1.3%), muscle soreness (1.3%), back soreness (0.9%), joint pain (0.9%), and insomnia (0.9%). The incidence was 0.4% for other adverse events, including poor sleep, anxiety, dry skin, armpit pain, dizziness, and chest pain. Most adverse events were grade I. Grade II adverse events rarely occurred, including bone soreness (0.4%), hot flushes (0.4%), morning stiffness (0.4%), and muscle soreness (0.4%) (Table 3). The above data suggested that leuprorelin (Boennuokang^®^) plus endocrine therapy might be a safe treatment for these patients.

**TABLE 3:** Adverse events.

Adverse events, n (%)	Total (N = 229)	Grade I	Grade II
Bone soreness	14 (6.1)	13 (5.7)	1 (0.4)
Hot flushes	8 (3.5)	7 (3.1)	1 (0.4)
Morning stiffness	3 (1.3)	2 (0.9)	1 (0.4)
Muscle soreness	3 (1.3)	2 (0.9)	1 (0.4)
Back soreness	2 (0.9)	2 (0.9)	0 (0.0)
Joint pain	2 (0.9)	2 (0.9)	0 (0.0)
Insomnia	2 (0.9)	2 (0.9)	0 (0.0)
Poor sleep	1 (0.4)	1 (0.4)	0 (0.0)
Anxiety	1 (0.4)	1 (0.4)	0 (0.0)
Dry skin	1 (0.4)	1 (0.4)	0 (0.0)
Armpit pain	1 (0.4)	1 (0.4)	0 (0.0)
Dizziness	1 (0.4)	1 (0.4)	0 (0.0)
Chest pain	1 (0.4)	1 (0.4)	0 (0.0)

## 4 Discussion

The main treatment goal of GnRH agonists in premenopausal women with HR^+^/HER2^−^ breast cancer is to suppress the production of sex hormones (Ma et al., 2024). This is achieved by initially stimulating and then desensitizing the GnRH receptors in the pituitary gland, leading to a decrease in the secretion of gonadotropins, including LH and FSH (Ortmann et al., 2002). A reduction in LH and FSH levels inhibits the activity of the gonads, ultimately lowering the levels of E2 (Ortmann et al., 2002). E2 plays a crucial role in breast cancer progression by binding to estrogen receptors, thereby promoting tumor cell proliferation, survival, and tumor growth (Lu et al., 2021). Several previous studies have elucidated the effects of GnRH agonists plus endocrine therapy on gonadotropins and sex hormones in premenopausal women with HR^+^ breast cancer (Kurebayashi et al., 2017; Lee et al., 2020; Masuda et al., 2011; Noguchi et al., 2016; Wu et al., 2021). For example, a previous study indicated that E2, FSH, and LH were lower after leuprorelin three- or 6-month depot plus tamoxifen in premenopausal women with HR^+^ breast cancer (Kurebayashi et al., 2017). Another study reported that E2 and FSH remained suppressed during 96 weeks of monthly or 3-monthly goserelin plus tamoxifen in premenopausal women with estrogen receptor-positive early breast cancer (Masuda et al., 2011). In line with these previous studies, we also discovered that E2, FSH, and LH were suppressed after leuprorelin (Boennuokang^®^) plus endocrine therapy in premenopausal women with HR^+^/HER2^−^ breast cancer. Although E2 was slightly increased from M6 to M24, its levels were still below 30 pg/mL. Maintaining E2 below 30 pg/mL indicates successful induction of a menopause-like state, which is related to better clinical outcomes in premenopausal women with HR^+^/HER2^−^ breast cancer (Corti et al., 2023). Therefore, leuprorelin (Boennuokang^®^) plus endocrine therapy could achieve a satisfactory ovarian function suppression effect, which may be beneficial in improving the prognosis of premenopausal women with HR^+^/HER2^−^ breast cancer.

GnRH agonists plus endocrine therapy could prolong survival in premenopausal women with HR^+^ breast cancer, according to previous studies (Francis et al., 2023; Francis et al., 2018). A previous study reported that the 8-year disease-free survival rate was 83.2% in premenopausal women with HR^+^ breast cancer receiving tamoxifen plus triptorelin, and it was 85.9% in those receiving exemestane plus triptorelin (Francis et al., 2018). Additionally, the disease-free survival rates at week 96 were 97.3% and 97.5% in premenopausal women with HR^+^ breast cancer receiving leuprorelin 6- or 3-month depot plus tamoxifen (Kurebayashi et al., 2017). In the present study, we reported the accumulating PFS rates during 10 years after leuprorelin (Boennuokang^®^) plus endocrine therapy in premenopausal women with HR^+^/HER2^−^ breast cancer. During the follow-up period, a total of 9 patients experienced disease recurrence, and the 10-year accumulating PFS rate was 91.7%. By comparison with the data of previous studies, leuprorelin (Boennuokang^®^) plus endocrine therapy had the potential to achieve satisfactory survival rates in premenopausal women with HR^+^/HER2^−^ breast cancer. Furthermore, we explored the factors related to PFS in premenopausal women with HR^+^/HER2^−^ breast cancer. It was found that comorbidity was independently related to shorter PFS. In our study, the comorbidities included several common conditions, such as Rathke’s cleft cyst, fatty liver, and thyroid nodules. However, only 11 patients had comorbidities, and the number of patients who experienced disease progression or death was small (n = 9). As a result, the statistical power of our analysis might have been constrained. Therefore, this finding required further validation in larger-scale studies.

Considering the history of systemic treatments might influence the outcomes of this study, we explored the relationship of the history of radiotherapy with PFS, gonadotropins, and sex hormones. It was found that the history of radiotherapy was not related to PFS, and changes in gonadotropins and sex hormones in premenopausal women with HR^+^/HER2^−^ breast cancer. Therefore, the history of radiotherapy might not affect the findings of this study. However, whether other systemic treatments like chemotherapy would affect the outcomes should be further investigated.

Common adverse events after GnRH agonists plus endocrine therapy in premenopausal women with HR^+^ breast cancer include hot flushes, nasopharyngitis, headache, back pain, insomnia, and musculoskeletal stiffness (Kurebayashi et al., 2017; Masuda et al., 2011; Noguchi et al., 2016). In this study, bone soreness (6.1%) was the most common adverse event, followed by hot flushes (3.5%), and musculoskeletal symptoms such as morning stiffness (1.3%) and muscle soreness (1.3%). No grade 3/4 toxicities were observed. The type of adverse events differed between our study and previous studies (Kurebayashi et al., 2017; Masuda et al., 2011; Noguchi et al., 2016). Additionally, compared to previous GnRH agonist studies, the incidence of back pain and insomnia appeared lower (Kurebayashi et al., 2017; Masuda et al., 2011; Noguchi et al., 2016). We speculated that these discrepancies might be attributed to the retrospective design of this study. The inherent biases associated with retrospective research could have led to inconsistencies in the types and incidence of adverse events compared with those reported in previous studies. Therefore, further prospective studies are required to validate the safety profiles of leuprorelin (Boennuokang^®^). Overall, the incidence of adverse events was relatively low in our study; no serious adverse events occurred, and most adverse events were mild and manageable, which did not affect patients’ quality of life and treatment adherence. Therefore, the safety profile of leuprorelin (Boennuokang^®^) plus endocrine therapy might be good in premenopausal women with HR^+^/HER2^−^ breast cancer. It should be acknowledged that long-term use of GnRH agonists may affect bone density (Henze and Stuckey, 2024). Therefore, studies with extended follow-up duration should be conducted to validate the long-term safety of leuprorelin (Boennuokang^®^).

According to the NCCN and ESMO clinical practice guidelines, OFS should be considered an adjunctive therapy in premenopausal women with HR^+^/HER2^−^ breast cancer (Gradishar et al., 2024; Loibl et al., 2024). In this study, leuprorelin (Boennuokang^®^) showed acceptable efficacy and safety in these patients. Therefore, leuprorelin (Boennuokang^®^) may serve as a potential OFS option for premenopausal women with HR^+^/HER2^−^ breast cancer. However, to support the wide clinical application of leuprorelin (Boennuokang^®^), further studies should consider investigating the cost-effectiveness of this drug in premenopausal women with HR^+^/HER2^−^ breast cancer.

This study has several important limitations. (1) Its retrospective, single-center design introduces potential selection and information bias, limiting the generalizability of findings. (2) The absence of a comparator group (e.g., patients receiving standard endocrine therapy without ovarian suppression or different GnRH agonists like goserelin or triptorelin). prevents definitive conclusions regarding the added benefit of leuprorelin (Boennuokang^®^). Therefore, further randomized controlled trials should be performed to validate the findings of this study. (3) The small number of disease progression or death events (n = 9) limits the statistical power of survival analyses. (4) Only the 28-day subcutaneous administration schedule was evaluated; therefore, the efficacy and safety of alternative dosing schedules remain unknown. (5) The median follow-up duration was only 38.1 months in this study, which was inadequate to assess the prognosis of premenopausal women with HR^+^/HER2^−^ breast cancer. Therefore, a study with a longer-term follow-up duration is required to assess the effect of leuprorelin (Boennuokang^®^) on PFS in these patients. (6) Genetic predisposition plays an important role in breast cancer progression, and tumor grading reflects the malignant degree and differentiation status of breast cancer cells. Therefore, these two factors might be potential confounders that would influence the findings of this study. (7) Further studies could consider obtaining data on RNA sequencing, proteomics, lipidomics, or metabolomics to provide valuable insights into the molecular state of patients with HR^+^/HER2^−^ breast cancer and contribute to the research progression of this cancer. (8) All included patients were in menopausal status at follow-up. Therefore, the impact of menopausal status on the efficacy of leuprorelin (Boennuokang^®^) should be investigated in further studies.

## 5 Conclusion

In conclusion, leuprorelin (Boennuokang^®^) combined with endocrine therapy effectively suppresses gonadotropins and E2 to postmenopausal levels and demonstrates encouraging long-term PFS rates with a favorable safety profile in premenopausal women with HR^+^/HER2^−^ breast cancer. However, given the study’s retrospective nature, lack of a comparator arm, and limited event rates, these results should be considered exploratory. Larger, prospective, randomized trials are necessary to confirm the therapeutic benefit and optimize the clinical use of leuprorelin (Boennuokang^®^) in this patient population.

## Data Availability

The original contributions presented in the study are included in the article/Supplementary Material, further inquiries can be directed to the corresponding author.
