# Supplementary figures and images for: Efficacy and safety of leuprorelin (Boennuokang®) plus endocrine therapy in premenopausal women with HR+/HER2− breast cancer
Source: Front Pharmacol. 2025 Jul 3;16:1594799. doi: 10.3389/fphar.2025.1594799 (PMC12267250; doi:10.3389/fphar.2025.1594799)

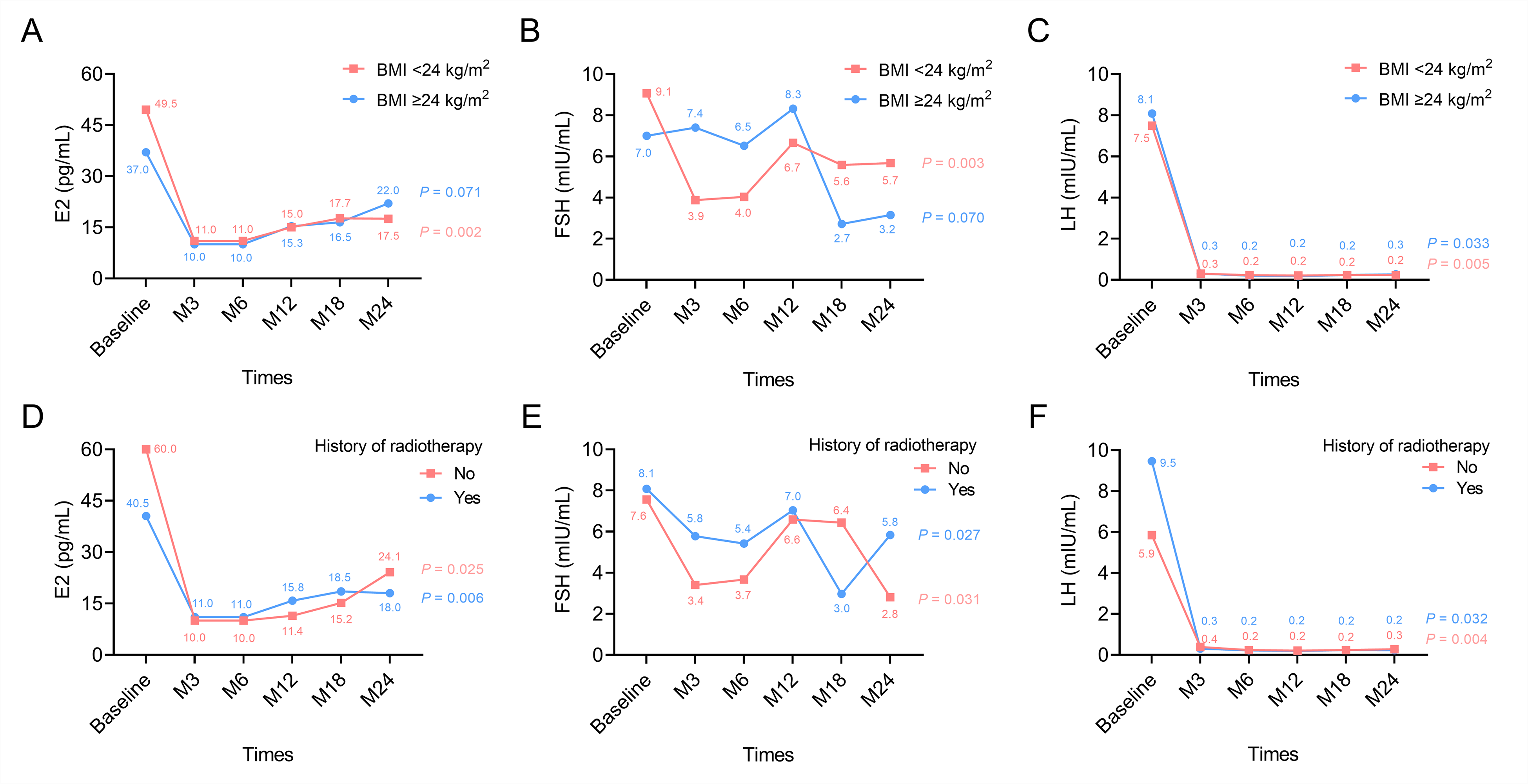

Supplement: Supplementary file 1 [file Image3.tif]

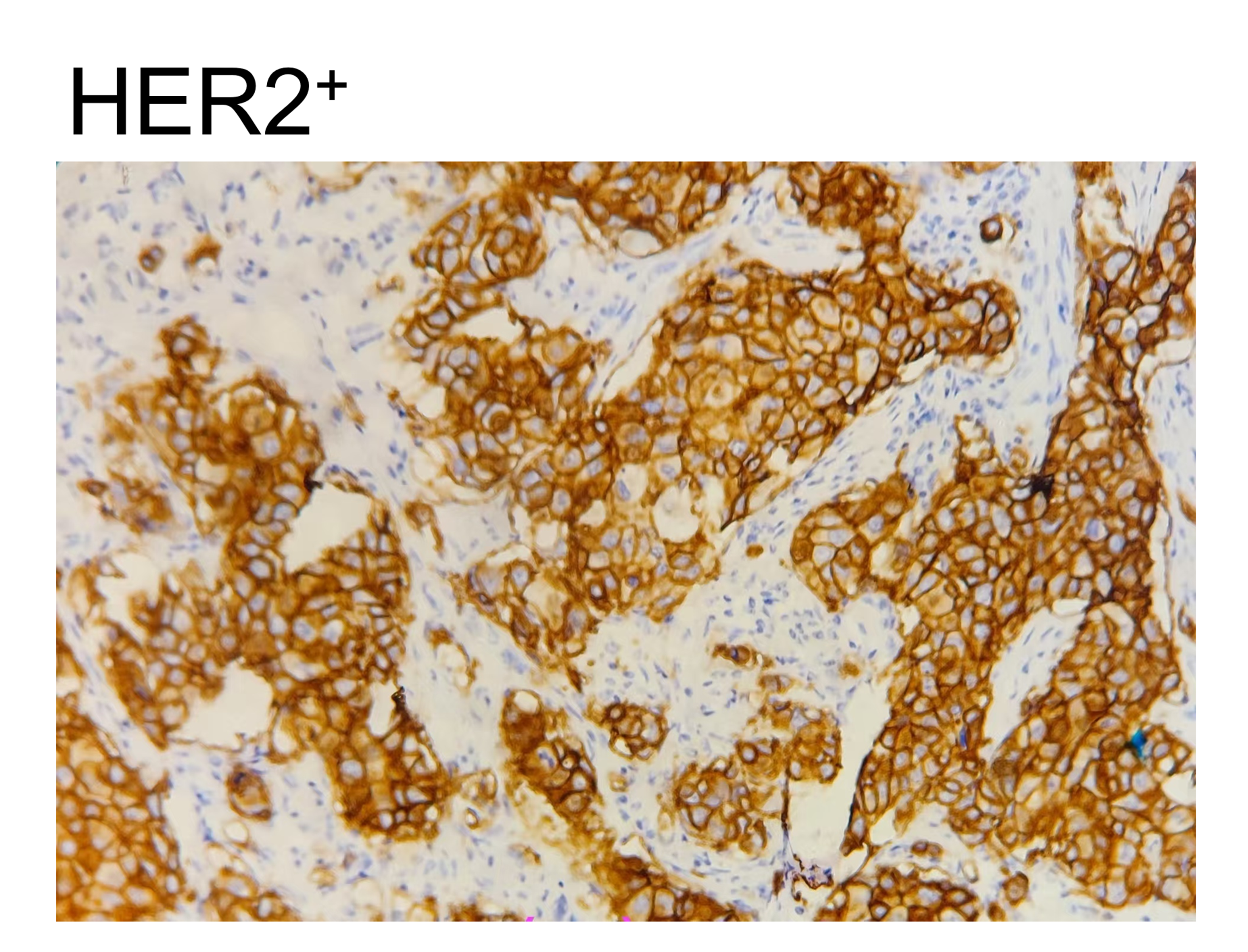

Supplement: Supplementary file 2 [file Image2.tif]

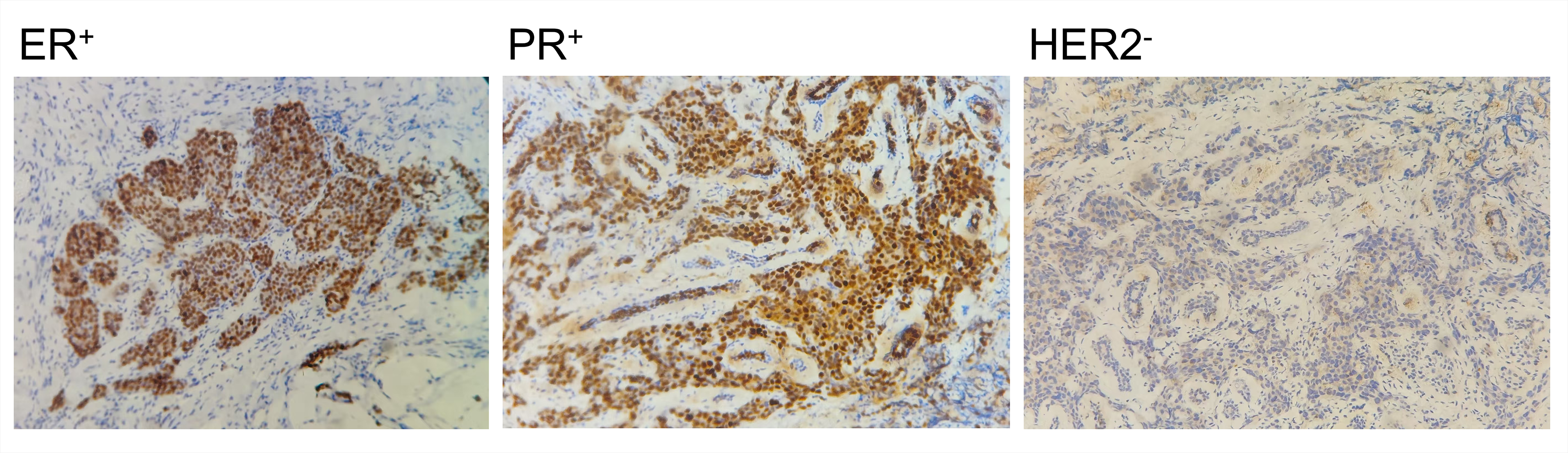

Supplement: Supplementary file 3 [file Image1.tif]
